# Design of an aluminium ion battery with a graphyne host: lowest volume expansion, high stability and low diffusion barriers†

**DOI:** 10.1039/d2na00058j

**Published:** 2022-08-17

**Authors:** Abhijitha V. G, Shashi B. Mishra, S. Ramaprabhu, B. R. K. Nanda

**Affiliations:** Condensed Matter Theory and Computational Lab, Department of Physics, IIT Madras Chennai 600036 India; Alternative Energy and Nanotechnology Lab, Department of Physics, IIT Madras Chennai 600036 India; Center for Atomistic Modelling and Materials Design, IIT Madras India nandab@iitm.ac.in

## Abstract

Commercialization of aluminium ion battery (AIB) requires limited volume expansion of the host cathode materials after AlCl_4_ intercalation, lower activation barrier, high theoretical specific capacity (TSC), cyclic durability and thermodynamic stability. Most of the carbon and non-carbon based cathode hosts explored so far failed to address the issue of volume expansion and there is a lack of clarity about thermodynamic stability. In this work, we employed multipronged first principles computational approaches on α- and γ-graphyne (GY) and showed that α-GY as a promising cathode host addresses each of the above concerns. Both α and γ-GYs provide ample space to accommodate more number of AlCl_4_ molecules leading to a high TSC of 186 mA h g^−1^ and open circuit voltages of 2.18 and 2.22 V, respectively. The absence of bond dissociation of AlCl_4_ and deformation of GY sheets at 300 and 600 K, as revealed by *ab initio* molecular dynamics (AIMD) simulation, indicates the stability of α- and γ-GY with adsorbed AlCl_4_. α-GY after intercalation shows a volume expansion of 186% which is the lowest among the cathode materials studied so far. The negligible expansion energy per unit surface area (∼0.003 eV Å^−2^) ensures the reversibility and hence cyclic durability of α-GY. Although the γ-GY shows a volume expansion of 249%, it is still promising. The NEB based diffusion study on monolayer and bilayer GY estimates the activation barriers to be (0.26, 0.06 eV) and (0.42, 0.16 eV) for α and γ phases, respectively. These values are either comparable to or lower than those of earlier reported cathode hosts.

## Abstract 2

Commercialization of aluminum ion batteries requires limited volume expansion of the host cathode materials after AlCl_4_ intercalation, lower activation barrier, high theoretical specific capacity, cyclic durability and thermodynamic stability.

## Graphical abstract

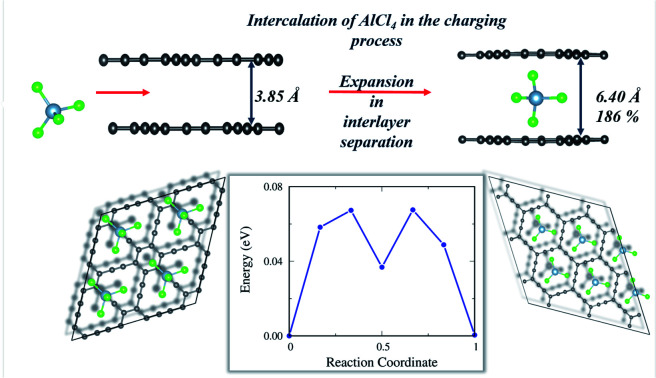

## 1 Introduction

In the recent past, aluminium based rechargeable batteries (AIBs) have received more attention from the worldwide research community as they are one of the most promising alternatives to the widely used Li-ion batteries (LIBs).^1^ Aluminium is an attractive anode material for secondary batteries, due to the fact that in comparison to the monovalent Li, K, and divalent Mg, Ca metals,^2,3^ Al can exchange three electrons (trivalency) in a redox reaction. As a result it can deliver approximately four times the volumetric capacity (8046 mA h cm^−3^) as compared to that of Li (2062 mA h cm^−3^).^4^ In addition, AIBs are preferred because of the large abundance, ease of handling and low flammability of Al. Furthermore, AIBs address the major drawbacks associated with the large scale application of metal ion batteries such as fabrication cost of electrodes, environmental hazards and safety issues like thermal runaway and dendrite formation.^5,6^

Though the Al metal anode in AIBs has great advantages over all other active metals, the commercialization of AIBs is still in its elementary stage. One of the main reasons is non-availability of suitable cathode materials for hosting the bulky AlCl_4_^−^ electrolyte anion governing the charging and discharging processes. The proposed reaction mechanism for AIBs is as follows:^7^1
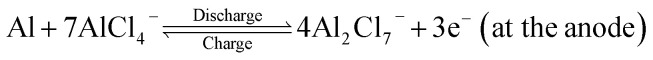
2
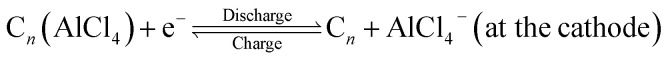


Intercalation of AlCl_4_ anions results in a large volume expansion of the cathode materials leading to cathode disintegration, which needs to be addressed for the commercialization of AIBs. In recent years, there has been steady progress in the development of new, suitable cathode materials for AIBs and a number of materials such as oxides, sulfides, polymers, Prussian blue analogues and various carbon based cathode materials have been explored.^5,8,9^ Among all the proposed cathode materials, carbon based ones (*e.g.* graphene and different forms of graphite) are the front runners as they exhibit a high operating voltage of ∼2.3 V and good cycle life up to ∼6500 cycles. However, these cathode materials suffer from low specific capacity (69 mA h g^−1^, ∼C_32_(AlCl_4_))^7^ and this is attributed to the small pore size, large atom density and inability to form lower stage (AlCl_4_ between all the layers of graphene) graphite intercalation compounds (GICs).^10^ Earlier experimental and theoretical studies^11,12^ on AIBs suggest that the existence of defects in the cathode structure improves their storage capacity. The reason given is that the dangling bonds in the defects show strong attraction towards AlCl_4_.^12^ In fact by introducing nano-voids in the graphene structure Yu *et al.*^13^ showed an improved storage capacity of 120 mA h g^−1^. These results motivated us to search for potential carbon based cathode materials having large pores that can accommodate a larger number of AlCl_4_ molecules with negligible volume expansion.

The 2D, non-natural and synthetically approachable carbon allotrope formed by the ‘yne’ modification of graphene, *i.e.* insertion of the acetylenic linkage (–C≡C–) between the carbon (C) atoms of graphene, is known as graphyne (GY).^14,15^ It is composed of both sp and sp^2^ hybridized C atoms in which the sp^2^ hybridized atoms form six membered C rings that are connected to each other by the acetylene linkage containing sp hybridized C atoms.^16,17^ Owing to the arrangement of the sp hybridized C atoms, multiple lattice types of GYs with different geometries are possible, such as α-, β-, γ-, (6,6,12)-GY and graphdiyne. The experimental synthesis of graphdiyne^18^ has accelerated the research work on other forms of graphyne. Recently, γ-GY was synthesized by a mechanochemical method using CaC_2_ and hexabromobenzene (PhBr_6_) as precursors.^19^ Though extended two dimensional structures of α-GY have not yet been synthesized, the already existing finite sized building blocks are promising.^20–23^ The theoretical study using cohesive energy, phonon dispersion and AIMD simulation established the stability of monolayer α-GY.^24^ GYs find their application in several areas of science such as H_2_ storage,^25,26^ gas sensing and desalination, *etc.* The large triangular and hexagonal rings in GYs provide enough space to accommodate a larger number of active metal and complex ions and thus they act as promising candidates for electrode materials in rechargeable batteries. With GYs as anode in metal-ion batteries, we can achieve a high storage capacity of 1117 mA h g^−1^ and, with the application of ∼12% strain, an ultrahigh TSC value of 2233 mA h g^−1^ for Li. Similarly, 558 mA h g^−1^ for Mg and 557 mA h g^−1^ for Na, with a low energy barrier of 0.24 eV for both Li and Mg, and 0.4 eV for Na-ion batteries can be achieved.^27–33^

**Fig. 1:**
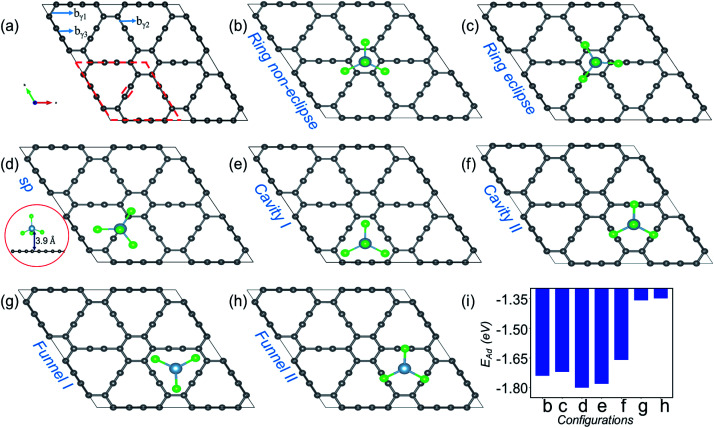
Site dependent AlCl_4_ adsorption on γ-GY. (a) Optimized 2 × 2 supercell of pristine γ-GY where different types of bonds, b_γ1_, b_γ2_ and b_γ3_, are marked, and the unit cell is highlighted with a red dashed line. (b and c) Represent AlCl_4_ adsorption at the hexagonal ring site in the non-eclipsed and eclipsed arrangements of Cl atoms. (d) AlCl_4_ adsorption on top of the sp-hybridized C atom in which the Al atom stays at 3.9 Å from the γ-GY sheet. The side view of this configuration is shown at the left end (red circled region). (e and f) Cavity site where the Cl atoms of AlCl_4_ are directed towards the sp^2^ and sp hybridized C atoms, and (g, h) represent their respective inverted funnel arrangement. (i) Adsorption energy calculated using eqn (3) corresponding to these seven configurations.

**Fig. 2:**
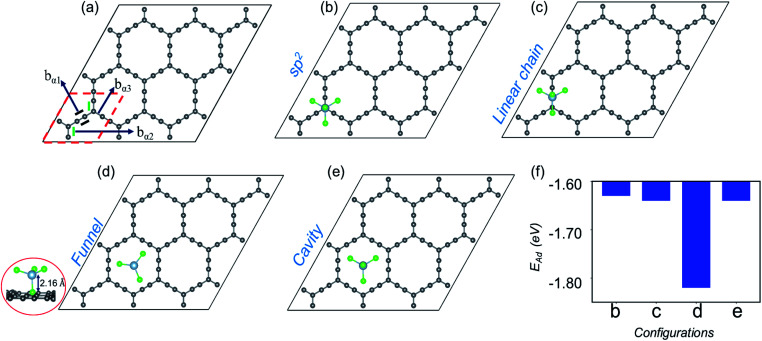
(a) Optimized structure of the 3 × 3 supercell of α-GY, where different types of bonds, b_α1_, b_α2_ and b_α3_, are marked and the unit cell is highlighted in red dashed lines. (b–e) Representation of the optimized structure for AlCl_4_ adsorption at sp^2^, cavity, funnel and linear chain sites, respectively and their adsorption energies in (f). The side view for the energetically favored funnel site is shown at the extreme left end.

In the present work, with the aid of DFT, CI-NEB and AIMD simulations, we establish that γ- and α-GY with large pores are potential cathode materials for AIB. Though the structures will be elaborated later, it is pertinent to mention at this point that while structurally the γ-GY consists of large triangular and small hexagonal rings as shown in Fig. 1, α-GY is formed of large hexagonal rings alone as shown in Fig. 2. We reveal that AlCl_4_ becomes adsorbed above the sp hybridized C atom chain in a standing configuration on γ-GY, while on α-GY it adsorbs preferentially at the hexagonal ring site in a funnel (inverted) configuration. From the electrochemical efficiency point of view, both γ- and α-GY exhibit high open circuit voltages (OCVs) of 2.22 and 2.18 V respectively and a superior TSC of 186 mA h g^−1^ for both materials. Furthermore, as inferred from the AIMD study, these GYs demonstrate excellent thermodynamic stability while adsorbing AlCl_4_ at 300 and 600 K. The NEB calculations predict that γ-GY exhibits a very low diffusion energy barrier of 0.06 eV, while α-GY shows a slightly higher barrier of 0.26 eV for the monolayer case and 0.16 and 0.42 eV for bilayer diffusion, respectively. Most importantly the volume expansion due to intercalation of AlCl_4_ in bilayer as well as trilayer α-GY is restricted to ∼186% which is remarkable when compared with the cases of graphite (262%), graphdiyne (270%) and hydrogen substituted graphdiyne (289%). Thereby, it addresses one of the major drawbacks related to AIBs.

## 2 Computational methods and models

The DFT calculations have been performed using the plane-wave pseudopotential code Quantum Espresso (QE) package.^34^ The exchange–correlation energy is approximated using the Perdew–Burke–Ernzerhof (PBE) functional. The ion–electron interaction is described by the Vanderbilt ultrasoft pseudopotentials and the energy cut-off for the wave functions is set at 45 Ry. The long range van der Waals interactions are included using DFT-D3 corrections.^35^ We performed geometrical optimization of the 2D hexagonal pristine and adsorbed GY structures using a slab model having a vacuum of 20 Å and the Brillouin zone was sampled with a 3 × 3 × 1 grid. The obtained lattice parameter for γ-GY is 6.87 Å which matches with the experimentally reported value of 6.90 Å.^19^ For α-GY the calculated lattice parameter is 6.95 Å which agrees well with previous theoretical reports.^36,37^ The diffusion energy barrier is calculated using the CI-NEB method of QE. The adsorption energy of AlCl_4_ on the GY surface is calculated using the following relation:3
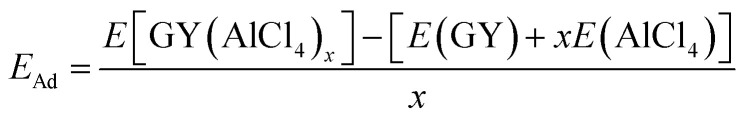
where *E*[GY(AlCl_4_)_*x*_], *E*[GY] and *E*[(AlCl_4_)] represent the total energies of GY (γ- and α) with adsorbed AlCl_4_, pristine GY surface and isolated AlCl_4_ respectively. The preferred tetrahedral geometry of AlCl_4_ with an optimized Al–Cl bond length and ∠Cl–Al–Cl bond angle of 2.14 Å and 109.4° (ref. 38) is considered for the adsorption study (see Fig. S1†).

The direction of charge transfer is analyzed through the charge density difference (CDD) calculated using the following expression.4
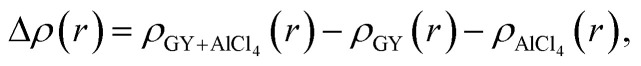
Here, *ρ*_GY+AlCl_4__ represents the charge density of GY with adsorbed AlCl_4_, while the pristine GY charge density is denoted by *ρ*_GY_ and *ρ*_AlCl_4__ represents the charge density of isolated AlCl_4_. The latter two components are calculated in the same coordinate space as that of the adsorbed surface, but with the removal of either components.

The thermodynamic study has been carried out using *ab initio* MD (AIMD) simulations in the NVT ensemble as implemented in VASP.^39^ The temperature of the system is fixed at 300 and 600 K using the Nosé–Hoover thermostat. In the present work, we have used 2 × 2 and 3 × 3 supercells respectively for γ- and α-GY. Initially the system is equilibrated for 5 ps followed by a production run for 25 ps with a time step of 1 fs.

## 3 Results and discussion

### 3.1 Structure and adsorption energy analysis

Fig. 1(a) shows the optimized structure of the 2 × 2 supercell of γ-GY having 48 carbon atoms with different types of bonds named b_γ1_, b_γ2_ and b_γ3_. The hexagonal ring is stabilized in sp^2^ hybridization with a bond length (b_γ1_) of 1.42 Å, similar to the atomic arrangement in graphdiyne.^38^ These rings are connected to each other through an acetylenic link (linear chain) containing sp hybridized C atoms and are composed of two different types of bonds, C(sp^2^)–C(sp) [b_γ2_] and C(sp)–C(sp) [b_γ3_], with bond lengths of 1.40 and 1.23 Å, respectively. The presence of both sp and sp^2^ hybridized C atoms results in triangular and hexagonal rings which facilitates AlCl_4_ adsorption.

As Cl atoms form the wings of tetrahedral AlCl_4_, different orientations are possible for AlCl_4_ on the γ-GY surface. We explored all possible adsorption sites and orientations of AlCl_4_ on γ-GY as shown in Fig. 1(b–h). The AlCl_4_ adsorbs on top of the hexagonal ring with non-eclipsed and eclipsed arrangements of Cl atoms, while in the latter one the Cl atoms are positioned along the linear chain of the C atoms (see Fig. 1(b and c)). Both these configurations lead to nearly equal adsorption strength as can been seen from Fig. 1(i). At the sp site, the Al atom of AlCl_4_ sits exactly above the sp hybridized C atom of the linear chain at a height of 3.9 Å from the γ-GY (Fig. 1(d)), while two of the Cl atoms of AlCl_4_ positioned above the triangular rings remain at a distance of 2.94 and 2.98 Å from the plane of C atoms, and another Cl atom positioned above the hexagonal ring stays at a distance of 3.19 Å. This configuration corresponds to the energetically favored arrangement of AlCl_4_. At the cavity site, the Cl atoms of the AlCl_4_ are directed towards the sp^2^ and sp hybridized carbon atoms respectively denoted as Cavity I and Cavity II in Fig. 1(e) and (f). The *E*_Ad_ plot shows that the Cavity I configuration is close to the energetically stable sp site configuration. This is due to the nearly same amount of charge transfer from the GY sheet to the AlCl_4_ molecule at Cavity I (∼0.76 e^−^). When the AlCl_4_ is placed in the inverted funnel arrangement at the cavity sites (Fig. 1(g and h)), we observed a significant reduction in the adsorption strength (Fig. 1(i)). This is because of reduced interaction between the Cl atoms of the AlCl_4_ molecule and nearby C atoms of the γ-GY sheet.

Now, moving on to the α-GY structure, where the C atoms show three different types of bonds, C(sp)–C(sp) [b_α1_], C(sp^2^)–C(sp) [b_α2_] and C(sp^2^)–C(sp) [b_α3_], respectively (Fig. 2(a)). The optimized bond length of b_α1_ is 1.23 Å, while b_α2_ and b_α3_ have equal lengths of 1.39 Å which is consistent with previous theoretical studies.^40–42^ A 3 × 3 supercell of α-GY allows us to examine the adsorption of AlCl_4_ in nine possible configurations, out of which four favorable sites are shown in Fig. 2(b–e) and the remaining are presented in Fig. S1.† In the sp^2^ and linear chain positions, the Al atom remains at a height of 3.86 and 3.89 Å from the nearest C atom, respectively (Fig. 2(b and c)). When the AlCl_4_ is positioned above the hexagonal cavity site, the Al atom remains at a distance of 3.74 Å from the α-GY sheet (Fig. 2(e)). Similarly, in the funnel configuration (Fig. 2(d)), the AlCl_4_ remains in the hexagonal ring, but the orientation is inverted as compared to the cavity site. Here, one of the Cl atoms remains exactly in the center of the cavity, while the Al atom and the other three Cl atoms remain at a height of 2.16 Å and 3.12 Å, respectively from the in-plane C atoms. The adsorption energy shows that the AlCl_4_ remains strongly bound to the α-GY sheet in the funnel configuration (see Fig. 2(f)). As a consequence, the charge transfer between the sp-hybridized C atoms and Cl atom becomes maximum which is discussed in the following section.

### 3.2 Electronic structure

**Fig. 3:**
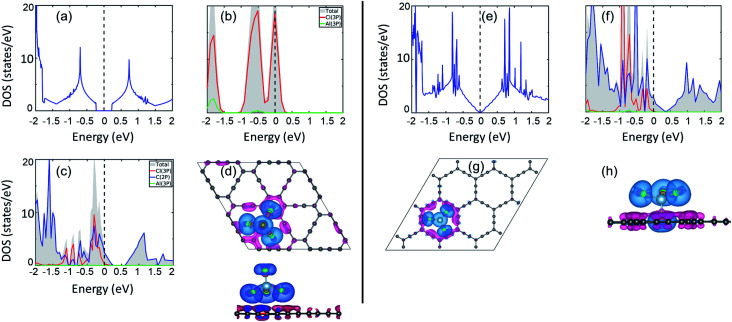
Electronic structure analysis. (a) Total DOS of pristine γ-GY; (b) total and partial DOS of isolated neutral AlCl_4_. (c) Partial DOS of γ-GY with adsorbed AlCl_4_ at the energetically most preferred sp-site; (d) top and side views of the charge density difference (CDD) plot for this stable configuration. (e) The DOS of the pristine α-GY sheet; (f) partial DOS for α-GY with adsorbed AlCl_4_ for the funnel configuration, and (g, h) corresponding top & side views of the CDD plot. Here, the blue and pink color charge contours represent the charge accumulation and depletion regions, respectively with an iso-value of 0.001 e Å^−3^. The Fermi energy is set to zero in the DOS plots.

Electronic structure lies at the core of what we observe microscopically. For the present case, change in the electronic structure of GY sheets and AlCl_4_ after adsorption holds the key to govern the electrochemical properties. The total density of states (TDOS) and partial density of states (PDOS) of pristine γ-GY and isolated AlCl_4_ are illustrated in Fig. 3(a) and (b), respectively. γ-GY is a semiconductor with a bandgap of 0.49 eV, which is similar to graphdiyne.^43^ It is in general true that the LDA/GGA functional underestimates the bandgap of a material. In case of graphdiyne, previous studies reported the increase in band gap from ∼0.45 eV within the LDA/GGA functional to 1.10 and 1.26 eV respectively using the more accurate and computationally expensive G_0_W_0_ and HSE06 functional.^44,45^ This increase in bandgap with the quasiparticle correction is attributed to the enhanced Coulomb interaction in the reduced dimension. However, as the focus of the present study is to examine the relative change in the electronic structure including the band gap, the GGA functional, which is computationally less expensive, is considered.

In the isolated AlCl_4_ molecule, the Cl 3p states dominate the Fermi level with a fraction of them remaining unoccupied because the three electrons provided by the Al atom are insufficient to form a closed shell configuration for each Cl atom (Fig. 3(b)). When AlCl_4_ is adsorbed at the most favored sp-site on γ-GY, AlCl_4_ as a whole acts as an electron acceptor, and charges are transferred from γ-GY to the Cl atoms. As a consequence, the Fermi level of γ-GY is shifted downwards as shown in Fig. 3(c). The direction of charge transfer is visualized through the CDD plot obtained by using eqn (4) (see Fig. 3(d)). The charge accumulation regions are represented by the light blue lobes and are centered on the Cl atoms, while the pink lobes show the charge depletion regions which are distributed on the planar γ-GY sheet.

Fig. 3(e) shows that the valence band and the conduction band of α-GY form Van Hove singularity and DOS vanishes at the Fermi level^43^ which is similar to what was observed in the case of graphene DOS.^46,47^ For the case of AlCl_4_ adsorption at the cavity site in the funnel (inverted) configuration on α-GY, we observed the same trend for DOS (Fig. 3(f)) and charge transfer (Fig. 3(g and h)) as in the case of γ-GY. The absence of a direct overlap between C(2p) and Cl(3p) states in PDOS for both γ- and α-GY indicates that the interaction between AlCl_4_ and C atoms can be attributed to a combined effect of van der Waals and ionic interactions.

Further, to quantify the magnitude of charge transfer, we performed Bader charge analysis of these adsorption sites for AlCl_4_ on γ- and α-GY. It is found that the total charge on the Al atom in both the cases remains unaltered (2.31|*e*|), while Cl atoms of AlCl_4_ receive charges from C atoms of γ- and α-GY, due to which AlCl_4_ as a whole entity acquires a net charge of 0.78 and 0.85*e*^−^. For AlCl_4_ adsorption at the sp site of γ-GY, the Cl atoms in the triangular and hexagonal rings receive an average charge of ∼0.2*e*^−^ from the surrounding C atoms. Meanwhile, in the funnel configuration on α-GY, all the Cl atoms remain closer to the GY sheet and as a result, each Cl atom receives ∼0.2*e*^−^ from the C atoms.

### 3.3 Coverage analysis

One of the distinguishing characteristics of a promising electrode in rechargeable batteries is high storage capacity, which is measured as a function of coverage. In this paper, we investigate the adsorption behavior and structural stability of γ- and α-GY with the increasing concentration of AlCl_4_ on their surface. After the first AlCl_4_ adsorption, there are several sites accessible for the second AlCl_4_ adsorption. The bulky size of the AlCl_4_ molecules, on the other hand, inhibits adjacent positioning, resulting in a minimum spacing of 3.5 Å between them.^38^

In previous reports, it is mentioned that adsorption on one side of the material gives a good approximation to estimate the specific capacity of an electrode.^48^ In all our calculations, we have considered the adsorption of AlCl_4_ on one side of GY, because here our aim is to use these as a reference for bulk or many-layer materials formed from these 2D counterparts. For example, if we consider a four-layered GY, for this particular system, AlCl_4_ can be intercalated in between the layers, above the top layer, and below the bottom layer. Now if we remove each layer one-by-one, the first three layers will have AlCl_4_ only on one side, while the bottom layer will have it on both sides. This indicates that single-sided adsorption is a better model for the capacity of a multilayer or bulk system than double-sided adsorption.

**Fig. 4:**
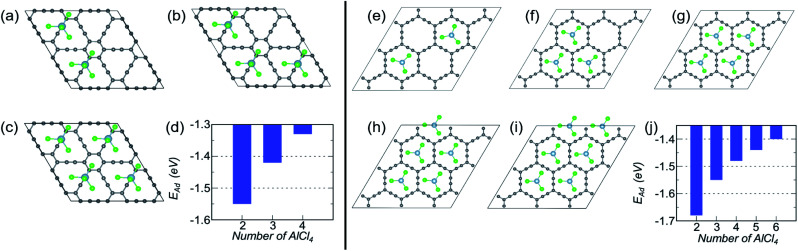
Coverage dependent adsorption study for γ- and α-GY. (a–c) Optimized structures of γ-GY with 2–4 adsorbed AlCl_4_ molecules and (d) their corresponding adsorption energies. (e–i) Representation of the most stable configurations for α-GY with 2–6 adsorbed AlCl_4_ molecules and (j) their respective *E*_Ad_.

Let us look into AlCl_4_ adsorption on γ-GY first. On a 2 × 2 supercell of γ-GY, we have illustrated the most stable configurations with two, three and four AlCl_4_ molecules in Fig. 4(a–c). The other possible configurations that were considered for each of these concentrations are presented in Fig. S3–S5.† The adsorption energy plot indicates that with the increase in the number of AlCl_4_ molecules, the adsorption strength decreases monotonically up to four AlCl_4_ molecules (Fig. 4(d)). With further increase in the concentration of AlCl_4_, increased interaction among the Cl atoms of the adjacent AlCl_4_ clusters occurs, resulting in the formation of Al_2_Cl_7_, as seen in graphdiyne.^38^ As a result, the TSC value for γ-GY determined using eqn (9) is 185.95 mA h g^−1^.

Owing to the larger in-plane area of α-GY as compared to γ-GY, it possesses more choices for increasing the number of adsorbed AlCl_4_ molecules on its surface. Fig. 4(e–i) show the stable configurations with 2, 3, 4, 5 and 6 AlCl_4_ molecules adsorbed on a 3 × 3 α-GY, while the configurations with weaker binding energy for each concentrations are shown in Fig. S6–S9.† Similar to the γ-GY supercell, the adsorption strength decreases monotonically with the increase in the number of AlCl_4_ molecules, which can be attributed to increased repulsion among the AlCl_4_ clusters (Fig. 4(j)). With further addition of AlCl_4_, agglomeration occurs, and hence, as with γ-GY, a TSC value of 185.95 mA h g^−1^ is obtained for AlCl_4_ adsorption on α-GY.

### 3.4 Thermodynamic stability

**Fig. 5:**
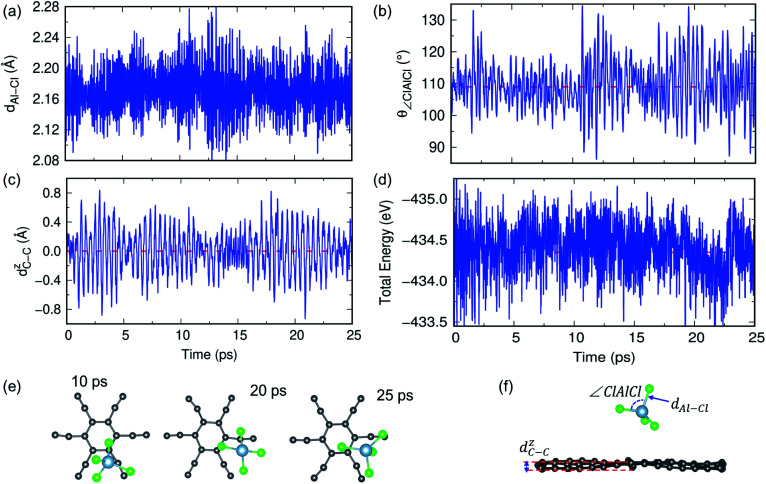
AIMD simulations for γ-GY with adsorbed AlCl_4_ at 300 K. (a and b) The time evolution of the Al–Cl bond length and ∠Cl–Al–Cl bond angle. The smaller fluctuation around the mean value indicates the stability of the tetrahedral geometry of AlCl_4_. (c) Buckling of the γ-GY sheet measured by the out-of-plane movement of C atoms. (d) The variation in total energy as a function of time. (e) Top view of snapshots of the structure at different time intervals. (f) Side view of the structure after 25 ps simulation and we have depicted various attributes of the MD simulation such as Al–Cl distance (*d*_Al–Cl_), Cl–Al–Cl angle (∠Cl–Al–Cl), and buckling of the GY sheet (*d*^Z^_C−C_).

In previous sections, we have discussed the suitability of GY for AIBs based on adsorption energy, electronic structure and coverage analysis. However, these studies are performed at 0 K, but for practical use of the material as an electrode, it is desired to perform the stability of the γ- and α-GY with adsorbed AlCl_4_ at finite temperature. Here, we performed AIMD simulations at 300 K and 600 K for the γ-GY+AlCl_4_ system, and the results are presented in Fig. 5 and S10.† The dynamical stability of the adsorption configuration at 300 K is investigated by several approaches such as analyzing the tetrahedral geometry of the AlCl_4_ molecule, buckling of the GY sheet, the energy of the total system and snapshots of the structure at different time intervals as shown in Fig. 5. The tetrahedral geometry of AlCl_4_ remains unaltered as observed from the minimal fluctuation of the Al–Cl bond length and ∠Cl–Al–Cl around their equilibrium values of 2.14 Å and 109.5°, respectively (see Fig. 5(a and b)). Next, we estimated the buckling of the GY-sheet by calculating the distance along the surface normal *z*-direction between two C atoms that undergoes maximum out-of-plane displacement and it is plotted as a function of time in Fig. 5(c). The buckling height is within the tolerance range (∼0.4 Å) and hence it implies the stability of the sheet. To further confirm the stability of the system, we plotted the total energy in Fig. 5(d) and it clearly agrees with the above observation that the system does not undergo any bond dissociation. Moreover, this is depicted by taking a few snapshots of the structure at different time intervals (Fig. 5(e and f)).

**Fig. 6:**
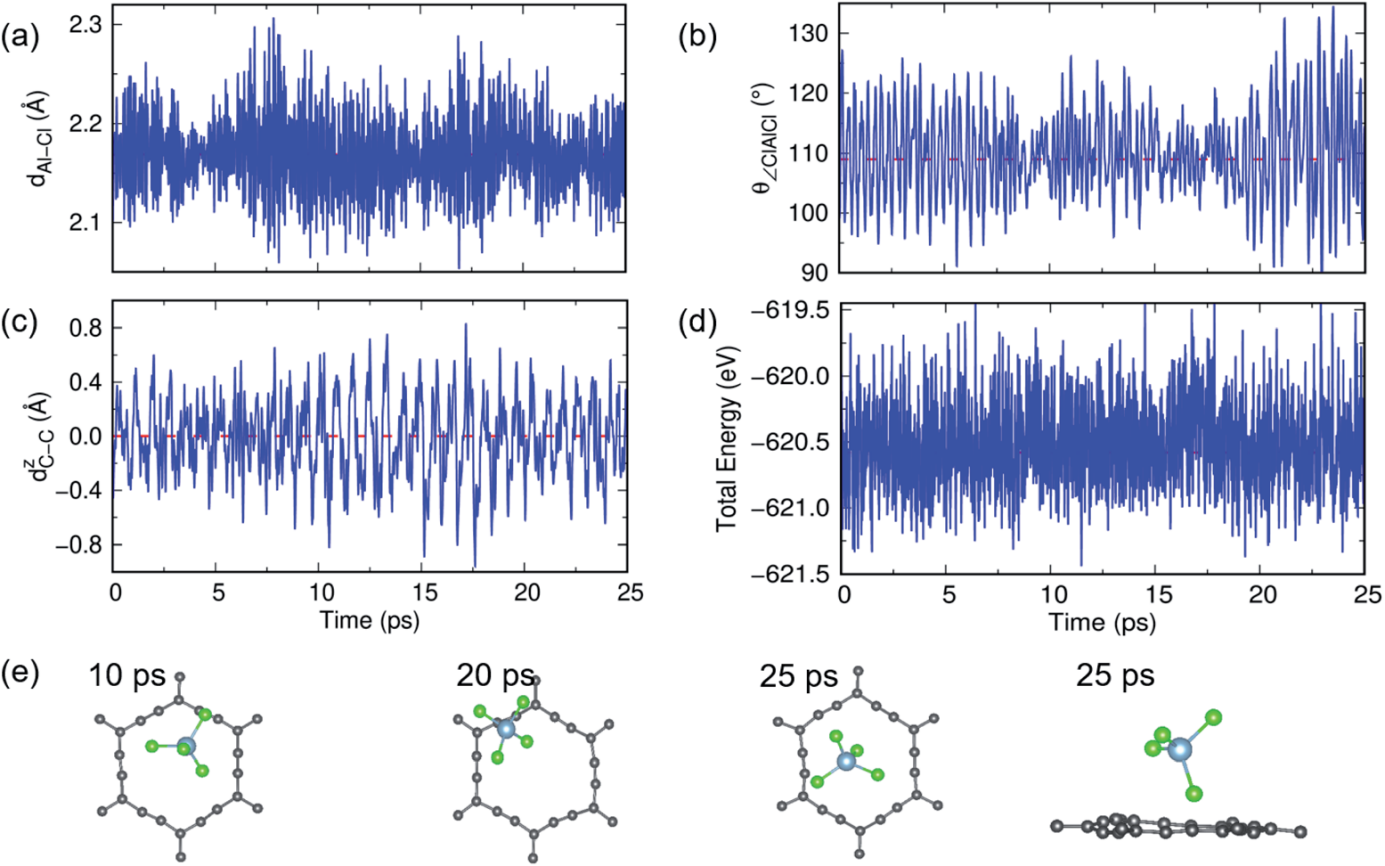
The structural stability study of α-GY with adsorbed AlCl_4_ at 300 K. (a and b) The dynamical fluctuation in Al–Cl bond length and ∠Cl–Al–Cl bond angle which indicates that the tetrahedral geometry of AlCl_4_ is maintained. (c) Buckling of the planar α-GY sheet and (d) the variation in total energy of the system as a function of time. (e) Top and side views of snapshots of the structure at certain time intervals.

A similar AIMD study has been carried out for AlCl_4_ adsorption on α-GY at 300 K and 600 K, and the results are shown in Fig. 6 and S11.† At 300 K, the Al–Cl bond length and ∠Cl–Al–Cl bond angle oscillate around the mean values of 2.17 Å and 109.83°, respectively, with low buckling of the α-GY sheet, and the total energy of the system equilibrates around −671.8 eV (Fig. 6(a–d)). The snapshots of time evolution of the α-GY+AlCl_4_ system at regular intervals are shown in Fig. 6(e) and it indicates that the Cl atom, which was close to the α-GY sheet in its equilibrium position, slowly moves upward and remains at a distance of ∼1 Å from the sheet without any structural deformations. The above study indicates that AlCl_4_ adsorption on GY sheets can be thermodynamically stable at room temperature.

### 3.5 Intercalation in multilayer graphyne

**Fig. 7:**
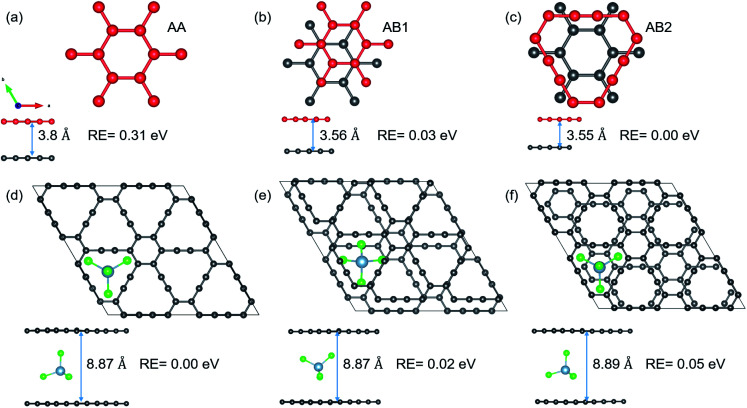
Top and side views of different stacking patterns of bilayer γ-GY: (a) AA; (b) AB1; and (c) AB2. The interlayer separation (*d*) and relative energy with respect to the preferred AB2 stacking are also included. (d–f) The optimized structures (top and side views) of the most stable intercalation configurations for each of the above-mentioned bilayer stackings. Total energy calculations reveal that AA stacked bilayer γ-GY is the most stable intercalation configuration with an interlayer separation of 8.87 Å.

As mentioned in the introduction, volume expansion, which causes cathode disintegration and a decrease in the TSC, is seen as a major impediment to AIB implementation in reality. We investigate the extent of volume expansion when the bulky AlCl_4_ is intercalated between bilayer GY. The optimized structures of the three most preferred stacking patterns for pristine bilayer γ-GY and corresponding intercalated configurations are presented in Fig. 7(a–f) along with their relative energy and respective interlayer separation. It is observed that the typical interlayer separation after intercalation increases to ∼8.8 Å (see the top and side views of Fig. 7(d and e)), which is similar to the case of graphdiyne, graphite,^10,38^ and hence the disadvantage persists.

**Fig. 8:**
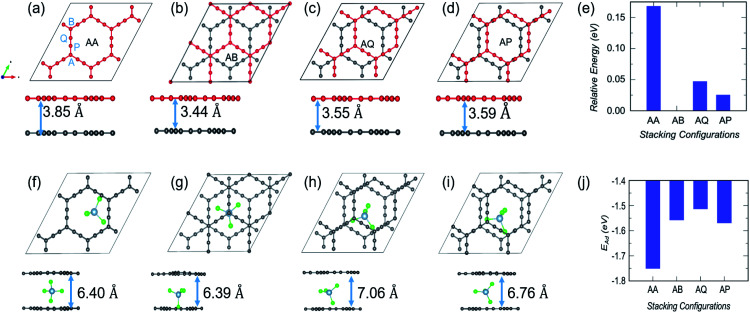
Top and side views of different stacking patterns of bilayer α-GY and corresponding intercalated structures. Bilayer stacking patterns such as (a) AA; (b) AB; (c) AQ; and (d) AP. The AA and AB stackings are self explanatory; however in AQ, A type C atoms from the top layer lie exactly over the bottom layer's Q type C atoms. A similar arrangement is followed up for the AP stacking. In each of the configurations the interlayer separation (*d*) is included. (e) The relative energy of these configurations with respect to the energetically most favored AB-stacked α-GY. (f–i) For each bilayer stacking, the top and side views of the optimized structures of the most stable intercalation site, as well as the corresponding interlayer separation following intercalation. (j) Variation in adsorption strength with stacking patterns.

In the case of α-GY, four possible configurations, AA, AB, AP and AQ, are examined as shown in Fig. 8(a–d). The C atoms are named A, P, Q and B according to the type of hybridization, *i.e.* sp^2^ or sp, and the configurations are described in the caption of Fig. 8. For the pristine bilayer α-GY, our optimization and total energy calculations reveal that the AB-stacking with interlayer separation of 3.44 Å is the lowest energy bilayer configuration. The relative energy of other optimized configurations is presented in Fig. 8(e). These inferences are in good agreement with earlier theoretical studies.^49,50^ However, as the energy difference between different stacking patterns is very small (< 0.2 eV) and with intercalation the preference of bilayer stacking patterns might switch,^51,52^ we have examined all four configurations for AlCl_4_ intercalation. The optimized structures of the most preferred intercalation site in each stacking pattern are shown in Fig. 8(f–i) and the adsorption energy study shows that the AA stacked α-GY intercalation structure (AA-AGIS) is energetically more favorable as compared to AB, AQ and AP (see Fig. 8(j)). Although in the pristine form, AB stacked bilayer α-GY is more stable, after intercalation with one AlCl_4_, AA-AGIS becomes more stable, which is similar to the case of Li intercalation in graphite where the most stable AB stacked structure changes to AA after lithiation.^51^ Interestingly in the case of α-GY, at the most preferred intercalation site (Fig. 8(f)), AlCl_4_ stays within the large hexagonal ring similar to the monolayer adsorption, leading to an inappreciable expansion in interlayer separation, which is discussed further in the following paragraphs. The expansion in interlayer distance (*d*) for AlCl_4_ intercalated AA stacked α-GY is estimated to be 6.40 Å, which corresponds to 166% of its pristine value (3.85 Å for AA stacked α-GY) and 186% with respect to AB stacked (3.44 Å) α-GY, and it is the lowest expansion in ‘*d*’ for the so far studied cathode materials of AIBs. The value of the expansion in case of graphite is 8.83 Å (262%),^10^ for bilayer graphdiyne it is 9.47 Å (270%)^38^ and for bilayer hydrogen substituted graphdiyne it is 9.07 Å (289%).^53^ As α-GY shows the lowest volume expansion, which we believe will facilitate the formation of lower stage (stage-1) AGIS, and will improve the efficiency of the AIBs by increasing the storage capacity. To substantiate further, we performed AIMD simulation at room temperature for AA-AGIS and the results are presented in Fig. S12.† We also examined intercalation of AlCl_4_ in trilayer α-GY (see Fig. S14†) and both studies reveal the stability of the intercalated structure. The mean value of interlayer separation in AIMD simulation is found to be 7 Å (181%), which is slightly higher in comparison with the value obtained from the DFT calculations and can be attributed to the thermal effects and in the case of trilayer intercalation the value is estimated to be ∼6.5 Å. To examine the reversibility of this expanded bilayer α-GY, we performed AIMD simulation on the optimized structure, Fig. 8(f), by removing the AlCl_4_ and the results indicate that within a short time span (3 ps), the structure regains its equilibrium AB stacked configuration (see Fig. 8(b)) with an interlayer separation of ∼3.4 Å (see Fig. S13†).

According to the reaction mechanism of AIBs, the AlCl_4_ intercalates into the cathode during the charging process and to accommodate bulky AlCl_4_, the interlayer separation of α-GY increases within a safety limit. Here we calculated the required expansion energy per unit area using the following expression:

5 *E*_exp_ = *E*_p_ − *E*_e_

where *E*_p_ and *E*_e_ represent the energy of pristine and expanded bilayer α-GY. Here we calculated the *E*_e_ by estimating the total energy of expanded bilayer α-GY after the removal of intercalated AlCl_4_. The *E*_exp_ is found to be 0.003 eV Å^−2^ for AA stacked α-GY which is very low compared to that of graphite (0.014 eV Å^−2^) and graphdiyne (0.006 eV Å^−2^).^53^ These results indicate the superiority of α-GY as a cathode material for AIBs over all other reported materials. We may note that if we consider full intercalation of AlCl_4_ (six AlCl_4_ molecules) within the 3 × 3 × 1 supercell, the volume expansion and the relative energy remains the same as shown in Fig. S15.†

### 3.6 Electrochemical properties of γ- and α-GY with adsorbed AlCl_4_

The open circuit voltage and theoretical specific capacity of the cathode are two important parameters in deciding the applicability of the battery in large scale storage devices. Higher values of both OCV (depending on electrolyte stability) and TSC are desired for battery operation. The OCV of a rechargeable battery depends on its underlying reaction mechanism. The electrochemical reactions in the AIB can be separated into two parts: (i) electrolyte reactions and (ii) electrode reactions, which are discussed in detail in our previous work.^38^ As AIB reaction mechanisms illustrate the active participation of electrolyte [EMIm(Cl)/AlCl_3_] anions AlCl_4_^−^ and Al_2_Cl_7_^−^, the reaction mechanism of AIBs in the presence of electrolyte cation EMIm^+^ can be written as:^7^6
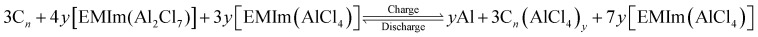


The effect of electrolyte cation EMIm^+^ on the OCV of the cell is negligible,^12,38^ hence, the above reaction can be reduced to7
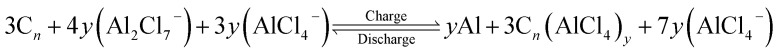


The voltage corresponding to reaction (7) is given by8
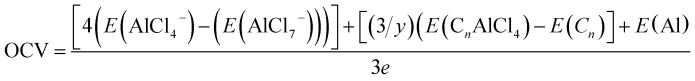
where *E*(*X*) represents the total energy of *X*. The factor 3 in the denominator indicates the involvement of three electrons in the electro-deposition of the Al atom. Due to non-availability of crystal structure for both anions, the value of [*E*(AlCl_4_^−^) − *E*(Al_2_Cl_7_^−^)] is calculated by optimizing each anion independently in a bigger cubic box. The OCV values calculated by using eqn (8) lie within the range of 1.94–2.41 and 1.92–2.35 V respectively for γ-GY and α-GY. In both cases the OCV values are lower compared to the electrochemical stability window of the electrolyte (2.45 V);^7,54^ this indicates that with γ- and α-GY as cathode in AIBs we can achieve a very high average OCV of 2.22 and 2.18 V for γ- and α-GY respectively, within the stability window of the electrolyte.

The theoretical specific capacity of the cathode is another important characteristic of a rechargeable battery. The TSC of any electrode in the rechargeable batteries is given by the following expression:

9 *C*_(γ,α-GY)_ = *nrF*/*M*_(γ,α-GY)_

where *n* is the valency of the ions involved in the electrochemical reactions, *r* is the number of moles of AlCl_4_ adsorbed per formula unit of γ- and α-GY, *F* is the Faraday constant (26 801.4 mA h mol^−1^), and *M*_(γ,α-GY)_ is the total molecular weight of γ- and α-GY per formula unit. As discussed in the coverage analysis section, γ- and α-GY can accommodate four and six AlCl_4_ molecules on 2 × 2 and 3 × 3 supercells, respectively. Hence, the value of TSC for γ- and α-GY estimated by using eqn (9) is ∼186 mA h g^−1^ for both γ- and α-GY.

### 3.7 Calculation of diffusion energy barriers

**Fig. 9:**
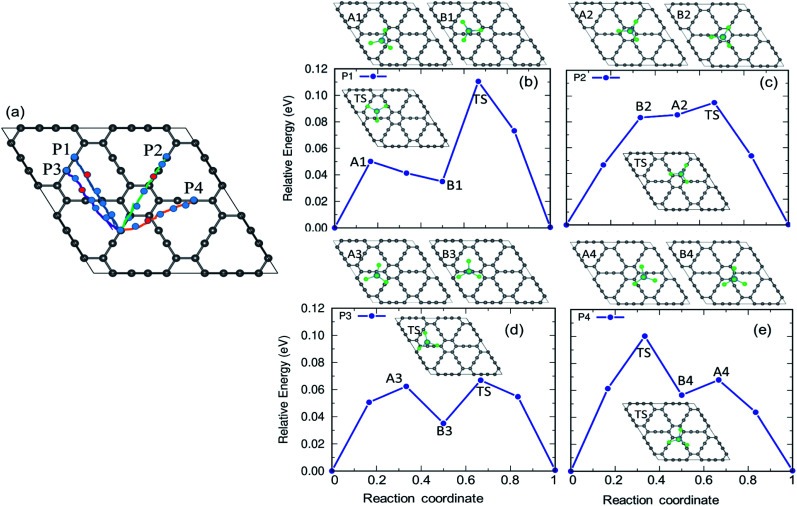
(a) The various diffusion pathways for AlCl_4_ on γ-GY: P1, P2, P3, and P4, and (b–e) the corresponding energy profile for each path. The inset depicts the transition states (TS) along these pathways. The points represented by the A and B series correspond to the local maxima and minima along the minimum energy path (MEP).

The charging and discharging capability of a battery is estimated by calculating the diffusion energy barrier. Here, we investigated four distinct paths for AlCl_4_ diffusion on γ-GY as shown in Fig. 9(a). AlCl_4_ diffuses through the triangular cavities with a minimum distance of 17.13 Å along path P1, with an estimated energy barrier of 0.11 eV (Fig. 9(b)). The corresponding transition state (TS) is located at the center of the triangular ring, where the Cl atoms of AlCl_4_ eclipse the surrounding sp-hybridized C atoms. Along path P1, we have also included local maximum A1 and minimum B1 in Fig. 9(b).

Along path P2, AlCl_4_ passes through the hexagonal ring and TS corresponds to a configuration where the AlCl_4_ orientation is exactly the opposite of the most stable arrangement (see the inset of Fig. 9(c)). With a path length of 15.36 Å, the energy barrier for path P2 is estimated to be 0.09 eV and the minima (A2) and maxima (B2) along P2 are shown in the top panel of Fig. 9(c). As A2 and B2 are similar to ring non-eclipsed and eclipsed configurations (see Fig. 1(b and c)), they lie higher in energy compared to the initial and final states of diffusion which is consistent with our adsorption energy analysis. The lowest energy diffusion path, P3, has a length of 14.57 Å and involves AlCl_4_ diffusion from a stable sp position to an adjacent sp site with an energy barrier of ∼0.06 eV (Fig. 9(d)). Along P3, AlCl_4_ crosses the linear chain at the middle and the TS is found at the corner of the triangular ring. The AlCl_4_ gets closer to the C atom chain and hexagonal ring along path P4 and it has a slightly higher energy barrier than path P3 (Fig. 9(e)). The minima and maxima corresponding to P3 and P4 are shown in the top panel of Fig. 9(d and e).

**Fig. 10:**
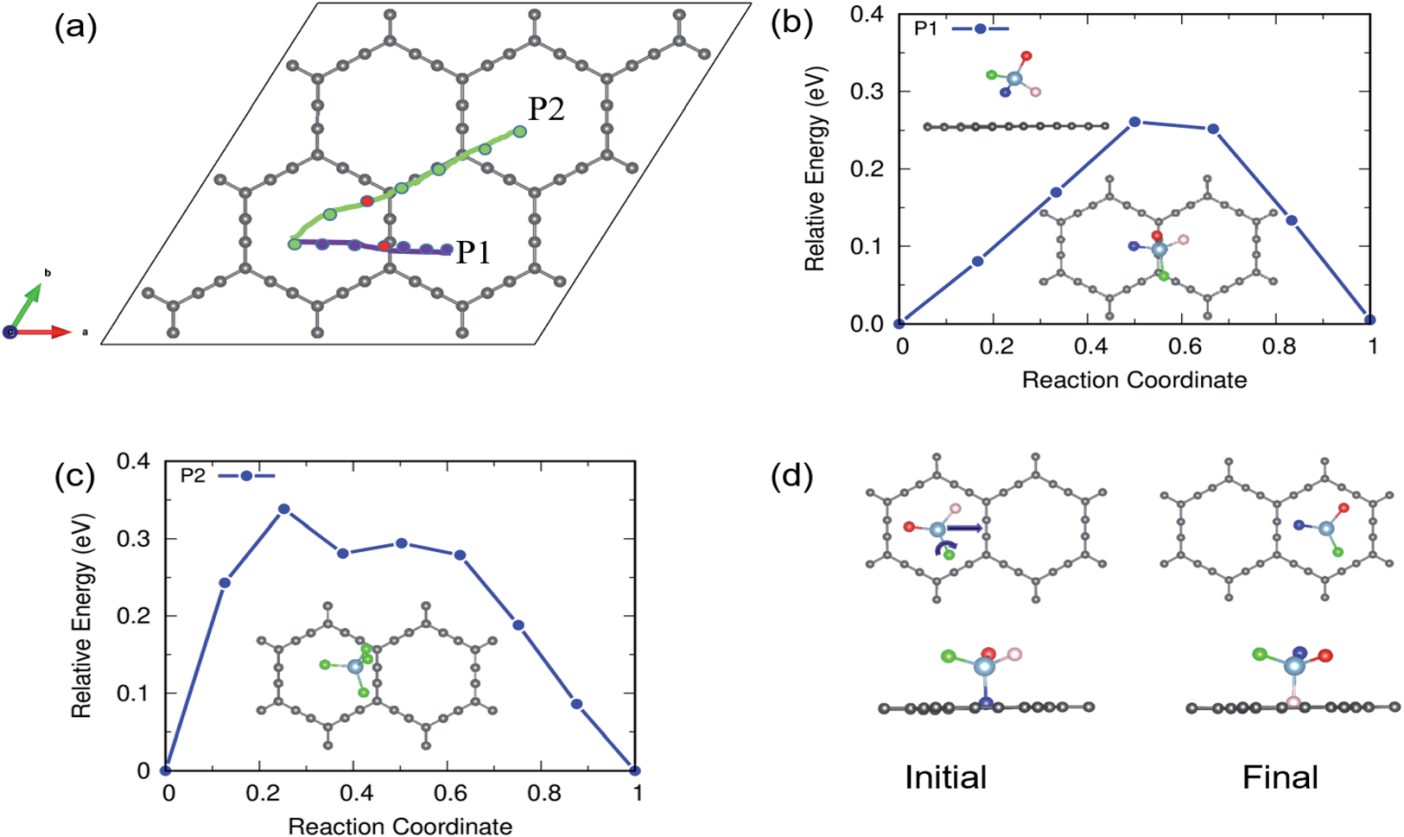
(a) Diffusion pathways of AlCl_4_ on α-GY. Diffusion energy barrier for (b) path P1, which runs across the linear chain, and (c) path P2, which runs parallel to it. In the inset, transition states near the linear chain of C atoms corresponding to each path are depicted. (d) Top and side views of path P1's starting and final states. To show the roto-linear motion of AlCl_4_ during diffusion along P1, the three Cl atoms in (d) are colored in three different colors (red, green, and peach).

In the case of α-GY, since the hexagonal rings are the most preferred sites for adsorption, only two diffusion paths are possible, namely, between nearest neighbor (path P1) and second neighbor (path P2) hexagonal rings as shown in Fig. 10. The AlCl_4_ diffuses across the C–C bond along P1 with a length of 19.05 Å, and the diffusion energy barrier is found to be ∼0.26 eV (Fig. 10(b)). Furthermore, the AlCl_4_ in the TS lies across the C–C chain, which agrees with our adsorption energy study (see Fig. 2(e)). The AlCl_4_ molecule moves from one hexagonal ring to another along the chain of C atoms with a diffusion barrier of 0.34 eV for path P2, which has a length of 28.52 Å (Fig. 10(c)). Although these values are greater than those of γ-GY, they are still two orders of magnitude lower than those of AlCl_4_ diffusion in other reported electrodes such as blue phosphorene.^55^ In the most stable adsorption configuration on α-GY, the funnel shaped AlCl_4_ lies at the center of the hexagonal ring (Fig. 2(c)). During diffusion, the AlCl_4_ molecule undergoes a roto-linear motion as shown in Fig. 10(d) and changes its configuration to the standing one at the TS (see the inset of Fig. 10(b)) and the same Cl atom moves upwards by a distance of 2.64 Å from the C atoms in the linear chain. The increased diffusion barriers on α-GY are mostly due to this change in configuration.

**Fig. 11:**
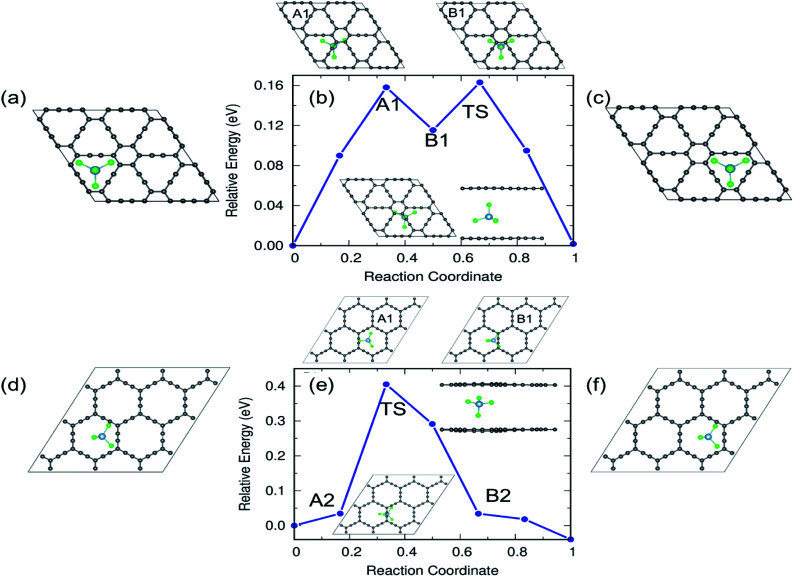
Reaction pathways and energy barriers for AlCl_4_ diffusion in bilayer γ- and α-GY. (a–c) Initial, energy profile and final configurations of the diffusion path for γ-GY. (d–f) Initial, energy profile and final configurations of the diffusion path for α-GY. The TS, intermediate maxima and minima are shown in the inset and top panel of the energy profile, respectively.

From the practical application point of view, diffusion of AlCl_4_ in multilayer GYs must be examined and here we restrict ourselves to the bilayer as they can be easily generalized to a larger number of layers. The results of the bilayer diffusion study for the most preferred sites in γ- and α-GY are shown in Fig. 11. The diffusion energy profile for the bilayer γ-GY with a straight line path between the initial (Fig. 11(a)) and final (Fig. 11(c)) triangular rings is shown in Fig. 11(b) and the corresponding energy barrier is 0.16 eV. The maxima (A1) and minima (B1) are presented in the top panel of the energy profile in Fig. 11(b), respectively. Similarly, Fig. 11(d and f) depict the initial and final configurations of the diffusion on α-GY. Fig. 11(e) shows the corresponding energy profile with a barrier of 0.42 eV and the top panel of the energy profile in Fig. 11(e) shows the maxima and minima corresponding to the path. Each path's TS is positioned between the linear chain of C atoms and is shown in the inset.

## 4 Summary and outlook

In summary, in order to design promising AIBs, we investigated the electrochemical performance of α- and γ-GY as potential hosts for AlCl_4_ using first principles calculations. The large triangular and hexagonal rings in the α- and γ-GY facilitate the adsorption of AlCl_4_ (six and four molecules) on their surface, leading to an appreciable TSC value of 186 mA h g^−1^ with OCV values of 2.18 and 2.22 V, respectively. Electronic structure analysis showed that the C atoms transfer a significant amount of charge to Cl atoms of AlCl_4_ and the bonding character is of ionic and van der Waals nature which enhance the cycling stability of the battery. Negligible buckling and preserved bonds in α- and γ-GY and AlCl_4_ demonstrate the stability of these structures at room temperature as inferred by the AIMD simulations. A multilayer intercalation study reveals the superiority of α-GY over the γ-GY as it shows only 186% expansion in the interlayer separation, lowest among the cathodes studied so far, while the latter one shows 249% expansion which is still lower than that of other carbon based hosts. The calculated diffusion barriers for AlCl_4_ on α- (0.26 eV) and γ-GY (0.06 eV) suggest the fast diffusivity of AlCl_4_ on these surfaces.

Table 1 contextualizes the present results in relation to the other reported AIB cathode hosts. As far as TSC is concerned, a large value is proposed for hydrogen-substituted graphdiyne (HsGDY), and black and blue phosphorene. In the case of HsGDY, the authors have demonstrated that on one side of the electrode we can place 20 AlCl_4_ clusters, based on adsorption energy analysis. But in a previous report,^10^ it has been stated that in order to reduce the repulsive interaction between the Cl atoms of adjacent AlCl_4_ clusters, a minimum distance of ∼3.5 Å must be maintained between them and in our previous work^38^ we have demonstrated that an adsorption study is a necessary but not sufficient tool for the accurate prediction of TSC. As the areal density of AlCl_4_ clusters increases, bulky Al_2_Cl_7_ clusters form and Cl_2_ gas is liberated. For black and blue phosphorene we can achieve a high capacity at a voltage of ∼3 V, but this is above the stability window of the electrolyte (2.45 V (ref. 7)) at which the decomposition of electrolyte occurs. Hence with α- and γ-GY, we can achieve 186 mA h g^−1^ TSC within the stability window of the electrolyte, while satisfying the above-mentioned distance criteria. In comparison to most of the cathode hosts mentioned in the table, the activation barrier for γ-GY is promising but for α-GY it is slightly higher. We observed that the diffusion energy barrier decreases as the height of adsorption increases for all electrodes except phosphorene allotropes due to their buckled nature, whereas all other electrodes considered are planar in nature. If we intercalate AlCl_4_ into the electrodes, most of them show a large expansion of interlayer distance except α-GY (186%). Meanwhile, γ-GY shows 249% expansion, which is still lower compared to the case of graphite, graphdiyne and HsGDY; therefore, we can address the cathode disintegration problem, a major roadblock for AIBs, with α-GY. We hope that the present work will spark new research initiatives for practical realization of commercially efficient AIBs.

**Table 1:** Comparison of various parameters such as TSC, OCV, diffusion energy barrier (*V*_B_), height of adsorption (*H*), and interlayer separation (*d*) for the bilayer of previously studied cathode materials for AIBs

Cathode material	TSC (mA h g^−1^)	OCV (V)	*V*_B_ (eV)	*H* (Å)	*d* (Å)	Ref.
γ-GY	186	2.18	0.06, ML	3.9	9	This work
			0.16, BL			
α-GY	186	2.22	0.26, ML	2.16	6.4	This work
			0.42, BL			
Graphite	69	2.3	0.03	4.7	8.83	10
Black phosphorene (ML)	432.29	2.94	0.19	4.68	—	55
Blue phosphorene (ML)	384.25	3.34	0.39	4.02	—	55
Hexagonal BC_3_	74.37	2.41	0.38	4.1	8.26	56
Graphdiyne (GDY)	186	2.3	0.08	4.2	8.68	38
HsGDY	456	2.15	—	4.0	8.21	53
C_3_N	178.7	0.67	0.03	4.3	8.69	8

## Author contributions

Abhijitha V. G.: conceptualization, investigation, data curation, formal analysis, writing – original draft, visualization. Shashi B. Mishra: investigation, formal analysis, writing – review & editing. S. Ramaprabhu: supervision, writing – review & editing. B. R. K. Nanda: conceptualization, formal analysis, supervision, resources, investigation, writing – review & editing, project administration, funding acquisition.

## Conflicts of interest

There are no conflicts to declare.

## Supplementary Material

NA-004-D2NA00058J-s001
