# Supplementary material for: Design of an aluminium ion battery with a graphyne host: lowest volume expansion, high stability and low diffusion barriers
Source: Nanoscale Adv. 2022 Aug 17;4(18):3870–82. doi: 10.1039/d2na00058j (PMC9470031; doi:10.1039/d2na00058j)
Supplement: NA-004-D2NA00058J-s001 [file NA-004-D2NA00058J-s001.pdf]

Electronic Supplementary Material (ESI) for Nanoscale Advances.
This journal is © The Royal Society of Chemistry 2022

Design of Aluminium ion Battery with Graphyne host: Lowest volume expansion, High stability and Low diffusion barriers

Abhijitha V G,[1,2] Shashi B. Mishra,[1] S. Ramaprabhu,[2] and B. R. K. Nanda*[,1,3]

[1]Condensed Matter Theory and Computational Lab, Department of Physics, IIT Madras, Chennai-600036, India

[2]Alternative energy and Nanotechnology Lab, Department of Physics, IIT Madras, Chennai-600036, India

[3]Center for Atomistic Modelling and Materials Design, IIT Madras, Chennai-600036, India

*E-mail: nandab@iitm.ac.in

I. Adsorption position on α-graphyne with lower adsorption strength

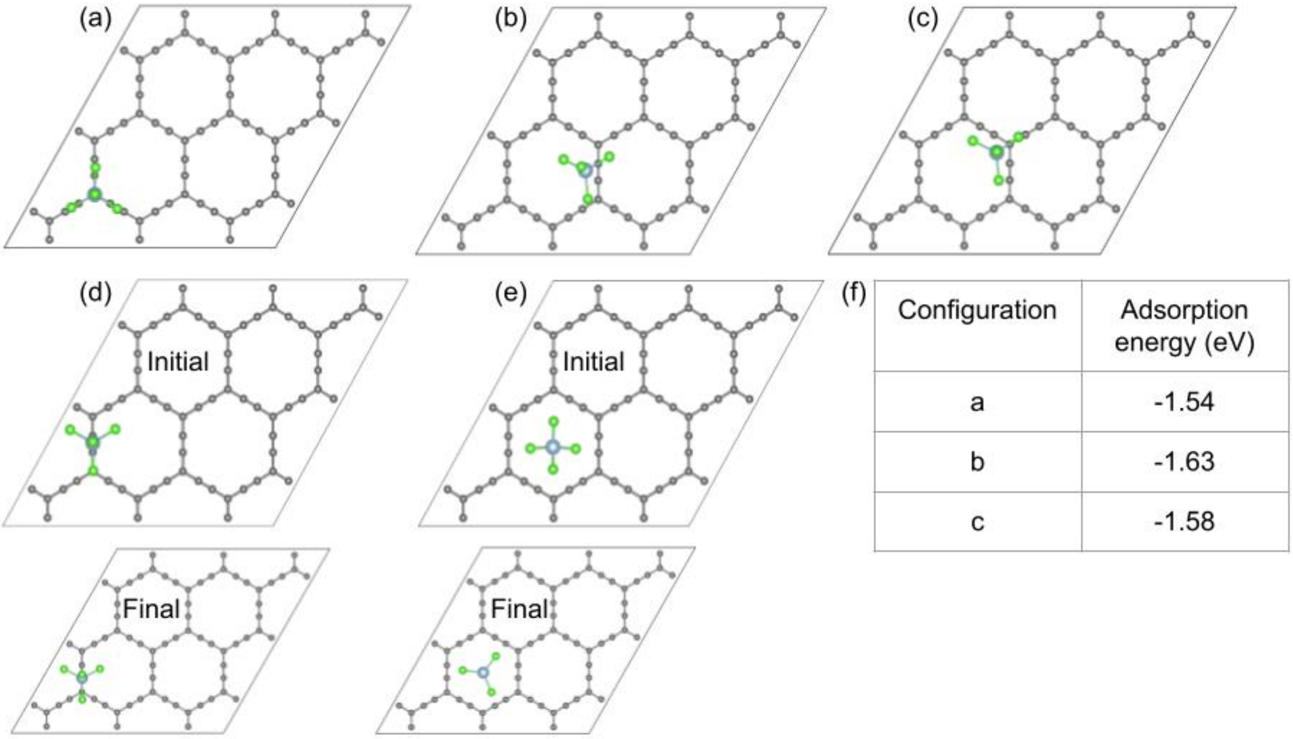

Configuration	Adsorption energy (eV)
a	-1.54
b	-1.63
c	-1.58

Figure S1: Optimized structures of few more adsorption configurations for $AlCl_4$ on α-GY. (a) Configuration in which Al atom of $AlCl_4$ remains above the sp^2 hybridized carbon atom, while Cl atoms remain above the linear chain; (b, c) Configurations with $AlCl_4$ closer to the linear chain of carbon atoms and corner of hexagonal ring, respectively. We showed two more starting configurations where (d) Al atom is positioned exactly above the mid-point of the linear chain and (e) Al is in the cavity with tilted arrangement for Cl atoms. These two configurations after structural relaxation led to the linear chain and funnel

configuration, respectively (see Fig. 2(c,d)) which are discussed in the manuscript. (f) Adsorption energy for unique configurations in (a-c).

II. Tetrahedral structure of $AlCl_4$

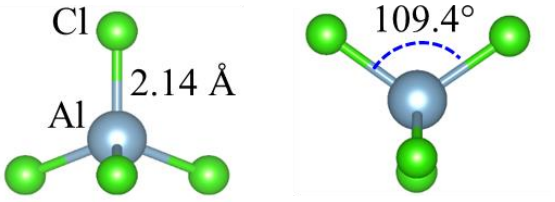

Figure S2. Optimized structure of tetrahedral $AlCl_4$ with Al-Cl bond length and ∠Cl-Al-Cl bond angle.

III. Coverage study for γ -graphyne

(a) Two $AlCl_4$ adsorption

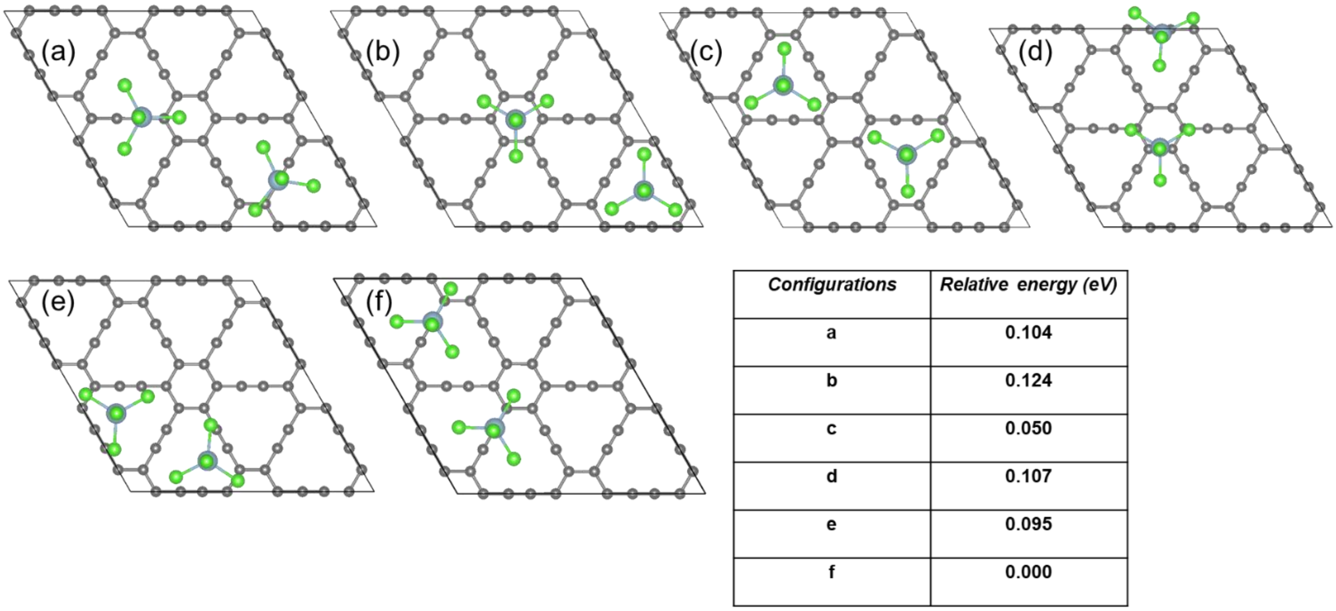

Configurations	*Relative energy (eV)*
a	0.104
b	0.124
c	0.050
d	0.107
e	0.095
f	0.000

Figure S3. (a-f) The adsorption of two $AlCl_4$ at different possible sites on γ-GY surface. The relative energy of each of the configurations with respect to preferred configuration (f), is tabulated. The most preferred site is discussed in detail in the manuscript.

(b) Three $AlCl_4$ adsorption

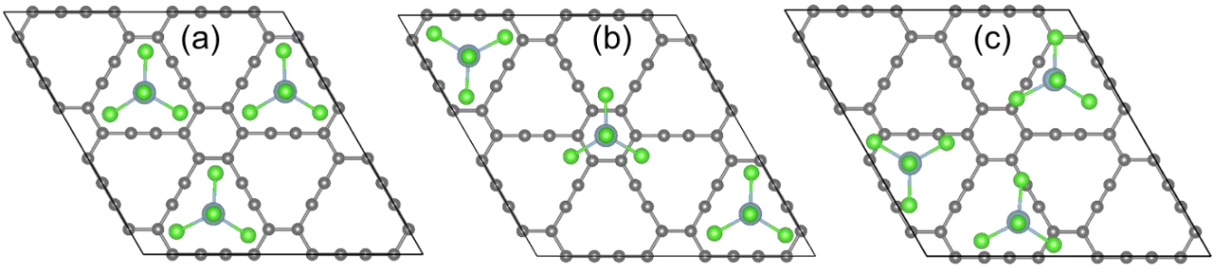

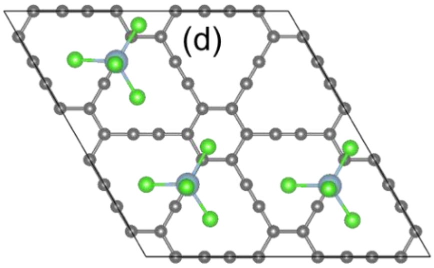

Configurations	*Relative energy (eV)*
a	0.32
b	0.20
c	0.24
d	0.00

Figure S4. Possible configurations for adsorption of three $AlCl_4$ on γ-GY and their relative energy where (a, b) correspond to $AlCl_4$ adsorption at triangular and hexagonal rings, (c) near to linear chain of carbon atoms, and (d) sp position. Their relative energy with respect to energetically preferred configuration (d) is tabulated.

(c) Four $AlCl_4$ adsorption

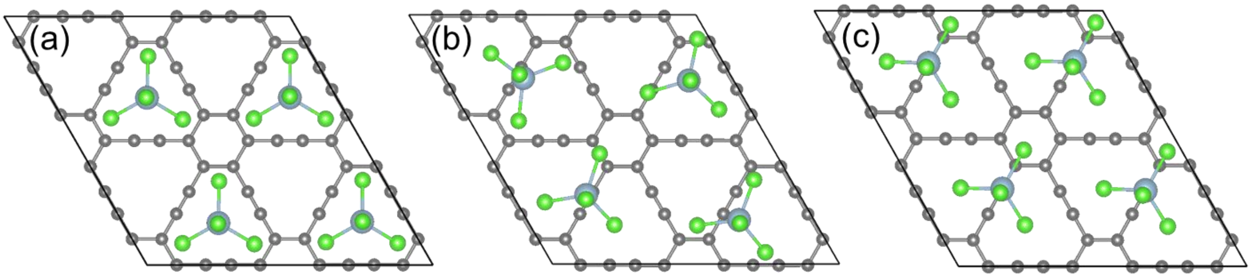

Configurations	*Relative energy (eV)*
a	0.23
b	0.56
c	0.00

Figure S5. (a-c) Various arrangements of four $AlCl_4$ on γ-GY and their relative energy with respect to the most preferred configuration (c).

IV. Coverage study for α-graphyne

(a) Two $AlCl_4$ adsorption

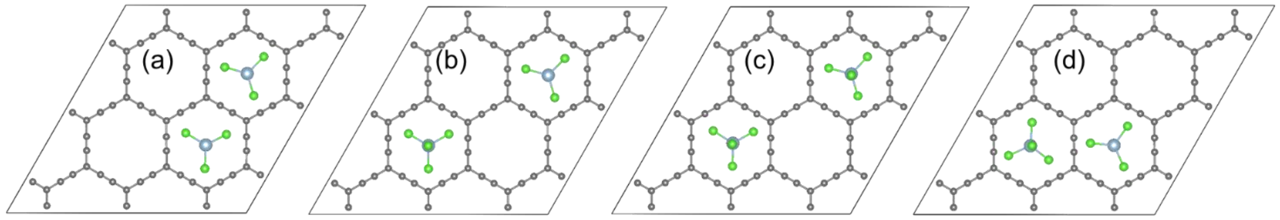

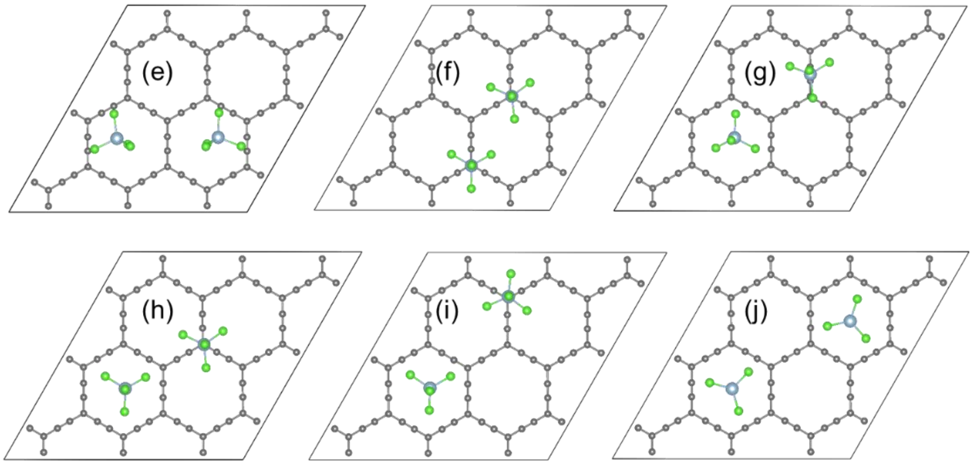

Configurations	Relative energy (eV)
a	0.07
b	0.20
c	0.40
d	0.26
e	0.23
f	0.51
g	0.43
h	0.46
i	0.43
j	0.00

Figure S6. Different arrangements of two $AlCl_4$ adsorption on α-GY and respective relative energies. (a-e) The configurations with $AlCl_4$ positioned in the hexagonal rings with both funnel and standing orientation of Cl atoms. (f) Configuration with both $AlCl_4$ positioned exactly above the sp^2 hybridised carbon atoms. (g-i) Arrangements with one of $AlCl_4$ placed above the linear chain and other in the ring site. (j) Stable adsorption configuration with two $AlCl_4$ and relative energies are calculated with respect to this configuration.

(b) Three $AlCl_4$ adsorption

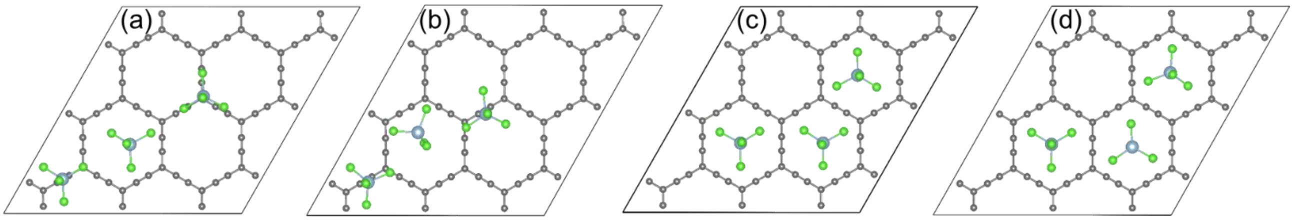

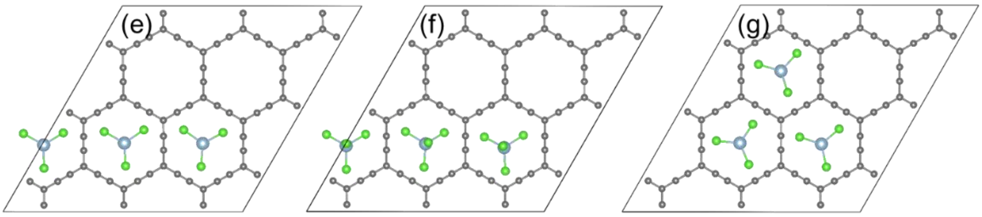

Configurations	Relative Energy (eV)
a	0.08
b	0.50
C	0.60
d	0.38
e	0.08
f	0.70
g	0.00

Figure S7. Arrangements of three $AlCl_4$ on α–GY. Configurations with $AlCl_4$ placed on (a) linear chain; (b) on linear chain but one of the $AlCl_4$ is flipped; (c) sp^2 carbon atom; (d) sp^2 carbon atom but with one of the $AlCl_4$ is flipped; (e-g) hexagonal rings positions and corresponding energy relative to energetically more preferred configuration (g).

(c) Four $AlCl_4$ adsorption

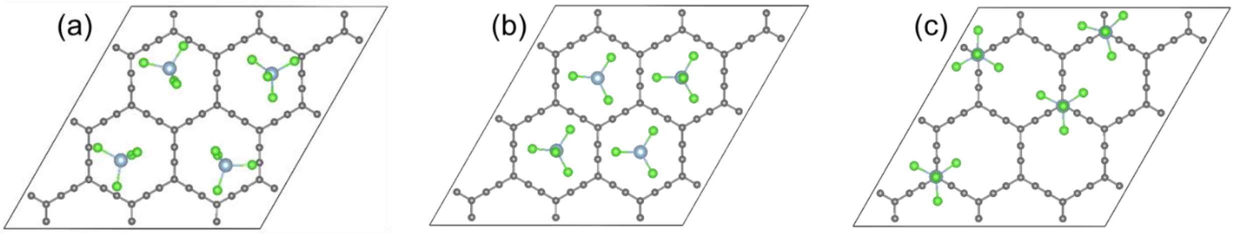

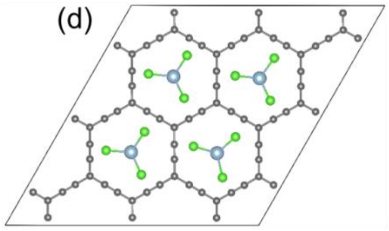

Configurations	Relative energy (eV)
a	0.44
b	0.43
c	0.93
d	0.00

Figure S8. Optimized structures of four $AlCl_4$ adsorbed α–GY and their relative energy. (a, b) Configurations with $AlCl_4$ placed in the hexagonal rings with funnel and standing arrangement of Cl atoms. (c) Configuration with $AlCl_4$ placed on the sp^2 carbon atoms, and (d) hexagonal ring which is energetically preferred site. The relative energy with respect to (d) is tabulated.

(d) Five and six $AlCl_4$ adsorption

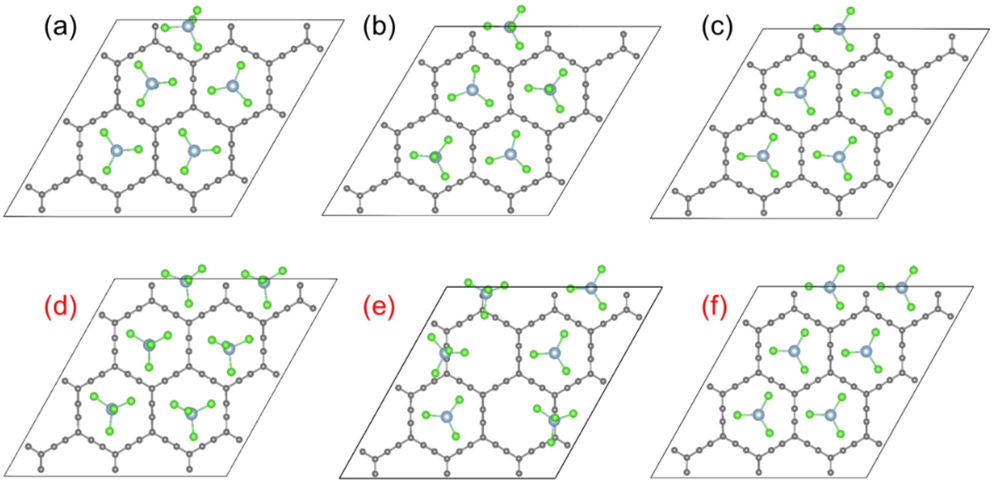

Configurations	Relative energy (eV)
a	0.12
b	0.70
c	0.00

Configurations	Relative energy (eV)
d	1.49
e	0.72
f	0.00

Figure S9. Other possible arrangements for five and six $AlCl_4$ adsorption on α–GY. (a, b) Five $AlCl_4$ adsorption in the hexagonal rings with both standing and funnel arrangement of Cl atoms. Similarly (d, e) represents the same for six $AlCl_4$ adsorption. (c) & (f) Lowest energy configuration for five and six $AlCl_4$ adsorption, respectively.

V. AIMD simulation of $AlCl_4$ adsorbed γ-GY at 600 K

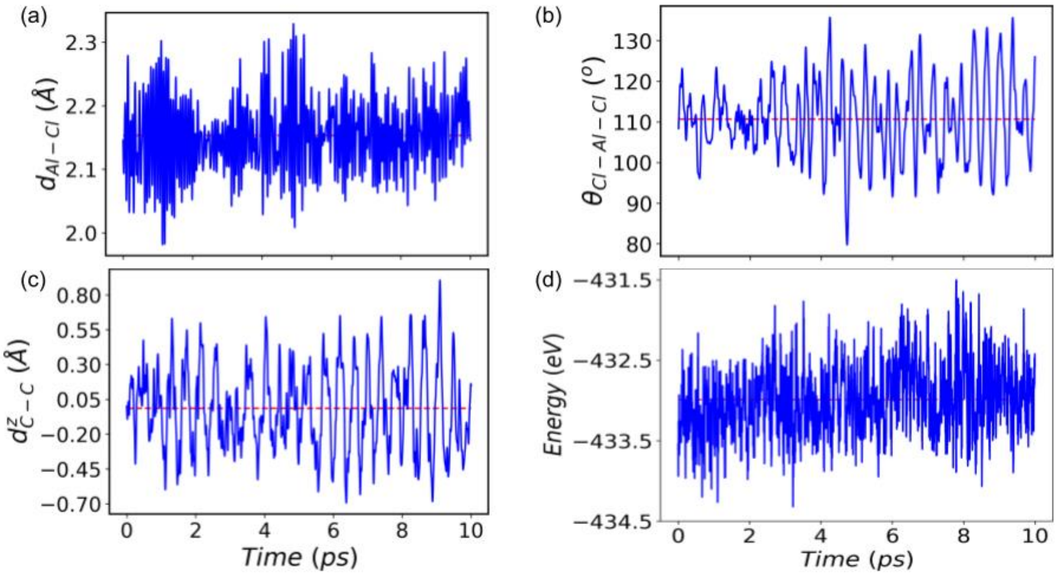

Figure S10. AIMD simulation for $AlCl_4$ adsorbed γ-GY at 600 K. (a, b, c & d) Variation in the Al-Cl bond length, ∠Cl-Al-Cl bond angle, out of plane movement of C atoms of γ-GY sheet and total energy of the system (γ-GY +$AlCl_4$).

VI. AIMD simulation of $AlCl_4$ adsorbed α-GY at 600 K

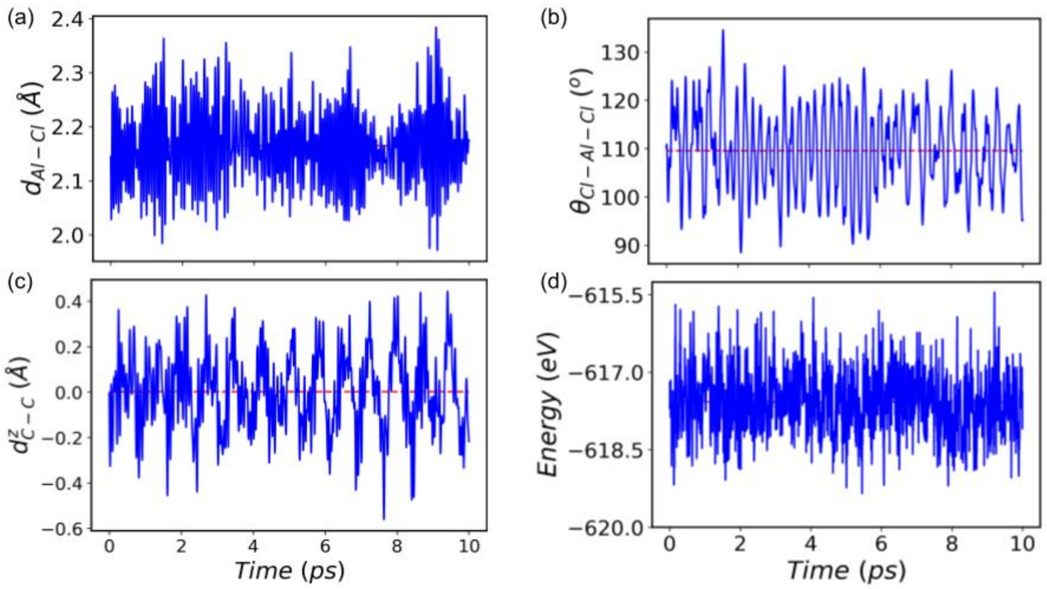

Figure S11. AIMD simulation for $AlCl_4$ adsorbed α-GY at 600 K. (a, b) Variation in the Al-Cl bond length and ∠Cl-Al-Cl bond angle. (c, d) Out of plane movement of C atoms of α-GY sheet and total energy of the system (α-GY +$AlCl_4$).

VII. Structural and cyclic stability of AA-AGIS

(a) Variation in the interlayer separation for $AlCl_4$ intercalated bilayer α-graphyne

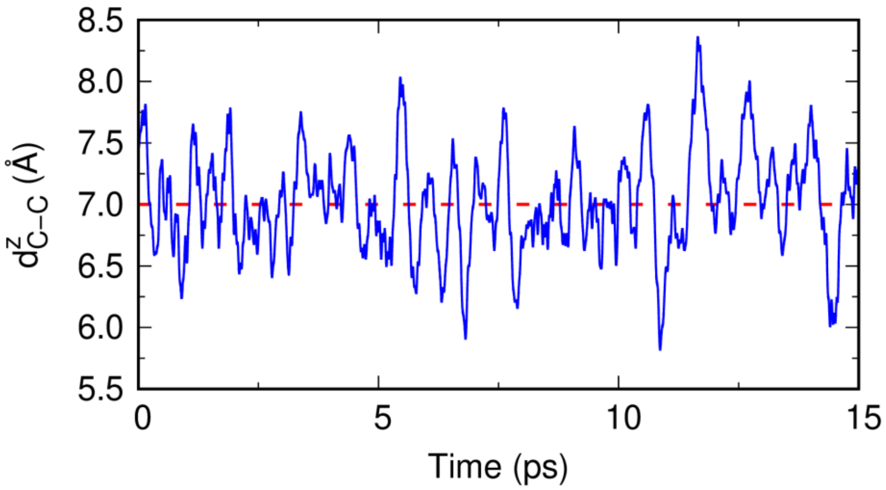

Figure S12. The variation of interlayer distance ‘*d*’ for AA-AGIS.

To validate our claim of lowest volume expansion of bilayer α-GY, we plotted variation in interlayer separation after intercalation obtained from AIMD simulation in Fig. S12. The result indicate that '*d*' varies about a mean value of 7 Å, which is slightly higher in comparison with DFT result and can be attributed to thermal affects, hence proves the structural stability of AA-AGIS.

(b) Cyclic stability of bilayer α-GY

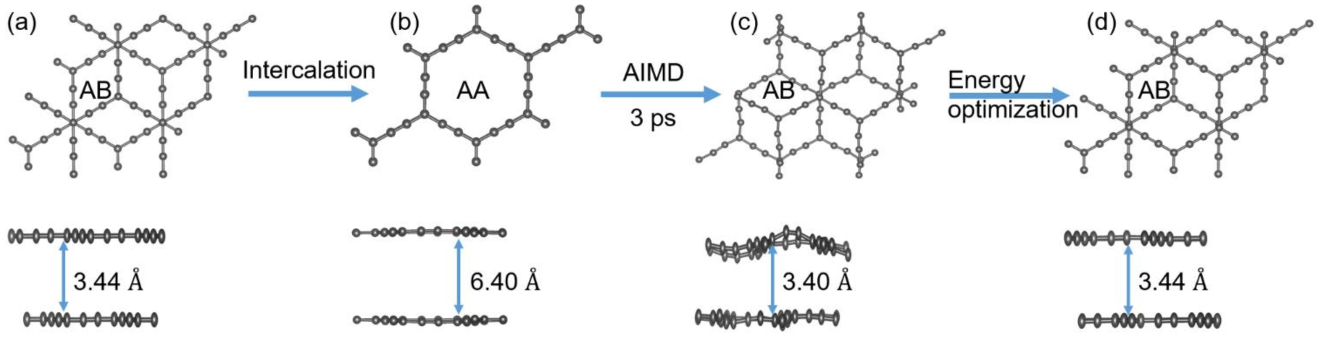

Figure 13. Top and side view of the Initial and final structure during the AIMD simulation. AIMD simulation is performed on the optimized $AlCl_4$ intercalated AA stacked bilayer α-GY structure by removing $AlCl_4$. After 3ps, the system regains its pristine optimized structure (AB-stacking) with a negligible buckling in the structure.

VIII. Trilayer intercalation study

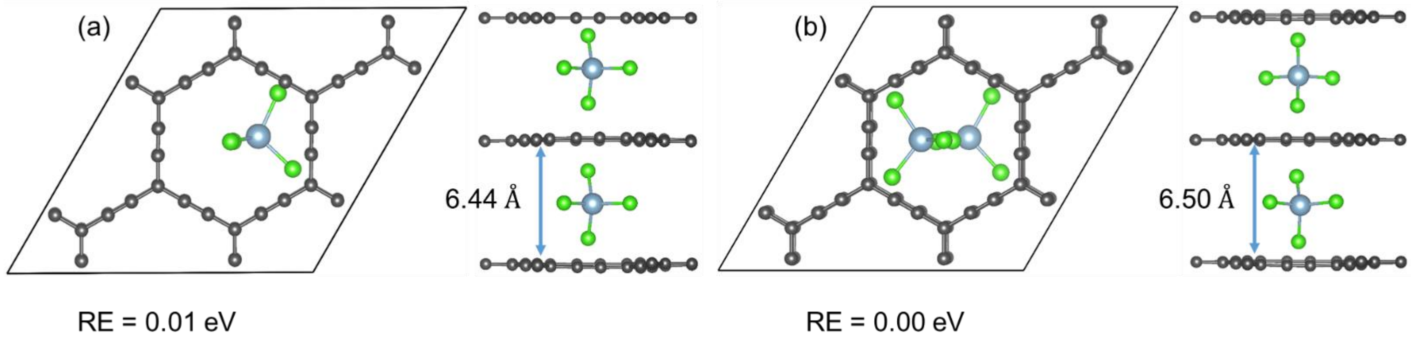

Figure S14. (a, b) Top and side view of optimized structures of $AlCl_4$ intercalation in three layered α-GY with two different arrangements for $AlCl_4$. Expanded interlayer separation and relative energy of the structures with respect to stable intercalation configuration (b) are also mentioned in each figures.

To study the multilayer effect on the interlayer separation and arrangement of intercalated $AlCl_4$, here we examine the $AlCl_4$ intercalated AAA-stacked trilayer α-GY. As Figure S7(a,b) depicts we have considered two distinct arrangements of the $AlCl_4$ for intercalation study. In Figure S7(a), two of the Cl atoms from both $AlCl_4$ are pointing towards the linear chain of carbon atoms similar to the bilayer intercalation, while in Figure S7(b), the Cl atoms from top and bottom gallery (empty space between any two layers of α-GY) points towards the linear chain in opposite direction. In both the cases, interlayer separation is ~6.5 Å which is in agreement with the bilayer separation (6.4 Å).

As both structures differ by 0.01 eV, it indicates the null effect of distinct arrangements of $AlCl_4$ on the total energy.

IX. Full Cell capacity of AIB with γ, α-GY cathode

The full cell capacity of the AIBs which is dependent on the quantity of the electrolyte considered is given by [1,2]

$$C_{full\text{-}cell\text{-}capacity} = \frac{C_C C_A}{C_C + C_A} \quad (1)$$

Where C_A denotes the TSC of anode (liquid electrolyte is considered as the anode which acts as a source for Al, $AlCl_4^-$ and $Al_2Cl_7^-$, C_C represents the TSC of cathode γ, α-GY.

The anode specific capacity (C_A) is given by:

$$C_A = \frac{Fq(x-1)}{xM_{AlCl_3} + M_{(EMIm)Cl}} \quad (2)$$

Where, x is the number of moles of $AlCl_3$ in the electrolyte, q (= 3/4) is the number of electrons involved in the electroactivity of one mole of $AlCl_3$ [2, 3]. M_{AlCl3} and $M_{(EMIM)Cl}$ represents the molecular weight of the electrolyte constituents.

The value of C_A calculated using Equation 1 for two extreme limits of 'x' i.e., x=1.3, 2 [3] is found to be 19 and 48 mAh/g, respectively. With these values of C_A 'x' = 1.3 and 2, respectively for γ, α-GY.

X. Bilayer intercalation study with increased $AlCl_4$ concentration

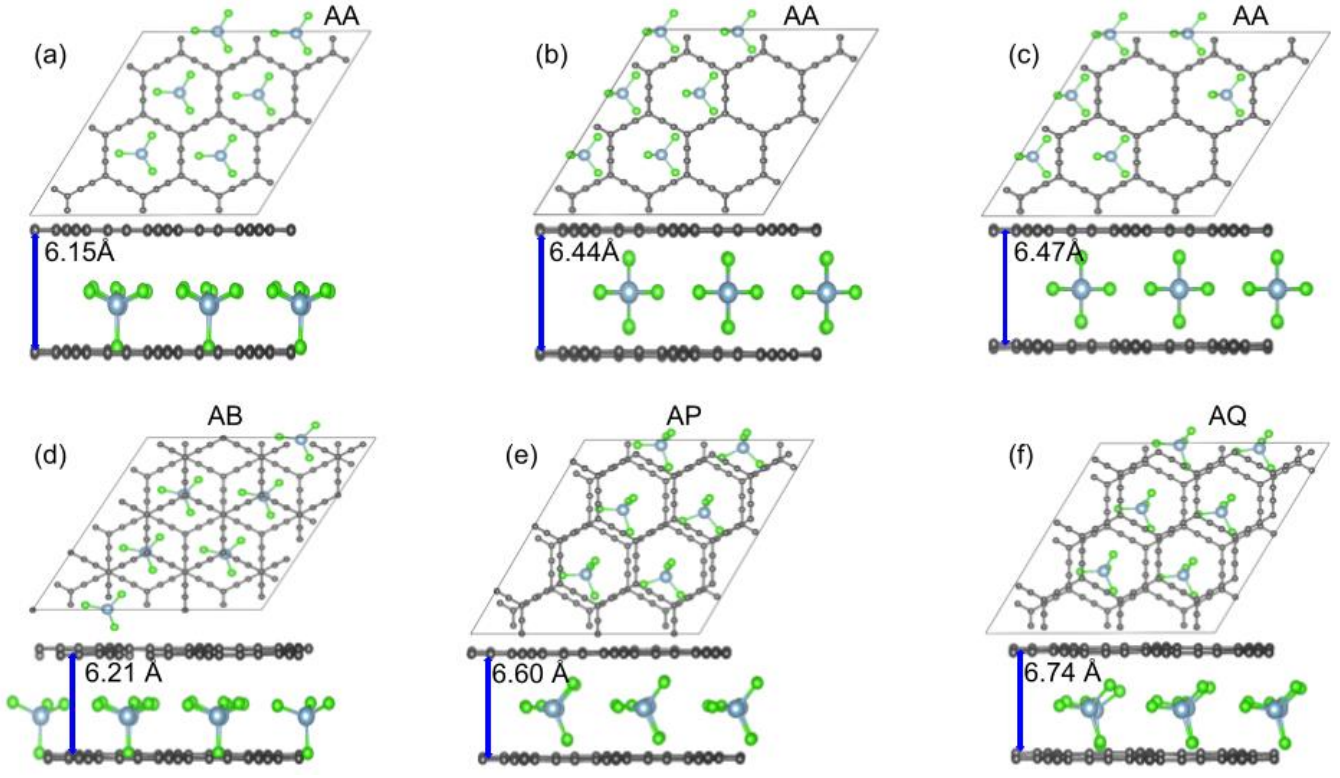

Figure 15: Intercalation of $AlCl_4$ in bilayer α-GY. (a-c) Represents the top and side view of intercalation in AA stacked bilayer α-GY with different arrangement for Cl atoms and the corresponding interlayer separations are also mentioned. (d-e) Represents the same for AB, AP and AQ stacked bilayer α-GY. These results agree with the one $AlCl_4$ intercalated 2 × 2 bilayer α-GY as presented in the manuscript.

REFERENCES

1) Kravchyk, K. V.; Seno, C.; Kovalenko, M. V. Limitations of Chloroaluminate Ionic Liquid Anolytes for Aluminum–Graphite Dual-Ion Batteries, ACS Energy Letters 5, 545–549 (2020).

2) S. Wang, K. V. Kravchyk, F. Krumeich and M. V. Kovalenko,ACS Applied Materials & Interfaces 9, 28478–28485 (2017).

3) B. Craig, T. Schoetz, A. Cruden and C. Ponce de Leon, Renew. Sust. Energ. Rev., 133, 110100 (2020).
